# What We Know About Team Dynamics for Long-Distance Space Missions: A Systematic Review of Analog Research

**DOI:** 10.3389/fpsyg.2019.00811

**Published:** 2019-05-15

**Authors:** Suzanne T. Bell, Shanique G. Brown, Tyree Mitchell

**Affiliations:** ^1^Department of Psychology, DePaul University, Chicago, IL, United States; ^2^Department of Psychology, Wayne State University, Detroit, MI, United States; ^3^School of Leadership & Human Resource Development, Louisiana State University, Baton Rouge, LA, United States

**Keywords:** team dynamics/processes, space exploration, astronaut, conflict, small sample, analog, over time changes, teams and groups

## Abstract

**Background:** To anticipate the dynamics of future long-distance space exploration mission (LDSEM) teams, research is conducted in analog environments (e.g., Antarctic expeditions, space chamber simulations), or environments that share key contextual features of LDSEM such as isolation and confinement. We conducted a systematic review of research conducted on teams in LDSEM-analog environments to identify which factors have been examined with quantitative research, and to summarize what the studies reveal about team dynamics in LDSEM-analog environments.

**Methods:** We used a comprehensive search strategy to identify research on teams that lived and worked together. Data on team dynamics were extracted where possible, and sources were coded for key contextual features. The data did not lend themselves to traditional meta-analysis. We used two approaches to summarize the data: a weighted averages approach when the study reported enough data to calculate an effect size, and descriptive figures when data across studies were directly comparable.

**Results:** Seventy-two sources met our inclusion criteria, yielding 253 effect sizes and 1,150 data points. Results from our weighted averages approach suggested that the team cohesion and performance relationship may be operating differently in isolated and confined environments than other teams that lived and worked together (e.g., military teams), and that, given the available data, we can say very little about the magnitude and direction of the relationship. Our descriptive figures revealed important trends: (a) team members in longer missions generally spent less social time together than shorter missions; (b) consistent team efficiency over time was typical, whereas decreased team efficiency over time was atypical; (c) by 40% of mission completion or 90 days, all teams reported at least one conflict, (d) commanders' written communication with mission control decreased in length over time, and (e) team mood dynamics did not consistently support the third-quarter phenomenon.

**Conclusions:** There are inherent limitations to our study, given the nature of the analog research (e.g., correlational studies, small sample size). Even so, our systematic review provides key insights into team dynamics in LDSEM-analog environments. We discuss the implications of our research for managing future space crews. Importantly, we also provide guidance for future research.

## Introduction

Extreme teams help to solve complex problems outside of traditional performance environments and have significant consequences associated with failure (Bell et al., 2018). As a type of extreme team, astronaut crews will be expected to live and work under psychologically and physically demanding conditions for future long-distance space exploration missions (LDSEMs), such as missions to Mars (Salas et al., 2015b). For example, LDSEM astronaut crews will be required to function effectively as a team in isolated and confined environments for up to 30 months (Human Exploration of Mars Design Reference Architecture [DRM] 5.0; Drake, 2009). LDSEMs will require crews to operate more autonomously as their communication with mission control (MC) will be delayed up to 22 min (DRM; Drake, 2009). Crewmembers will switch between periods of high and low workload, as well as between individual and team tasks. It will be necessary for the LDSEM crew to work together seamlessly for demanding team performance situations such as landing on Mars, keep conflicts manageable, and provide one another with social support as crewmembers deal with the stressors of prolonged space flight.

### Rationale

The National Aeronautics and Space Administration (NASA) and other space agencies seek to optimize team performance to minimize the risk of mission failure, and work with researchers from various scientific disciplines to prepare for future LDSEM missions. While meta-analytic investigations of important team relationships exist (e.g., team cognition, cohesion, composition, and performance), these investigations include traditional work team samples and findings may not necessarily generalize to the LDSEM context (Beal et al., 2003; Bell, 2007; DeChurch and Mesmer-Magnus, 2010; Bell et al., 2011). As such, researchers collect data in spaceflight and Earth-based analog environments, which are thought to mimic the challenges crews will encounter in LDSEM, to best design, prepare, and support future LDSEM crews and mission teams. Research on natural analogs examines teams that exist outside of research purposes; examples include polar stations in the Antarctic, where teams conduct scientific research while living in an isolated and harsh environment (e.g., Leon et al., 2011). Research in controlled analogs includes teams that exist specifically for research purposes; examples include teams in HI-SEAS, Human Exploration Research Analog (HERA) at Johnson Space Center, and the NEK facility at the Russian Academy of Science's Institute of Biomedical Problems (e.g., Ushakov et al., 2014; Binsted, 2015; Roma, 2015).

Analog settings share similar characteristics of LDSEMs expected to challenge crews and possibly impinge on team dynamics. As examples, analog crews live in a confined space (i.e., small living and working spaces with minimal privacy, physical discomfort), are isolated from others (i.e., limited interaction with others outside the crew, difficulty in communicating with family), are surrounded by a harsh physical environment (i.e., an environment in which survival is not possible without special equipment), have variable workload (i.e., a high and low volume of work at different periods), and have long-duration missions (i.e., the team works together for an extended period of time). Each analog may have its strengths and weaknesses given that not all of the environmental factors may be present in a particular analog. For example, crews in Antarctic stations experience physical confinement and isolation, but are typically isolated as smaller crews for shorter periods than is expected for LDSEMs. They also have environmental cues not available in spaceflight (e.g., daylight). Crews in space simulations (e.g., HUBES, SFINCSS) may experience isolation and confinement but are typically not surrounded by a harsh physical environment.

Research on teams in analog environments has a rich history. In fact, a number of factors (e.g., compatibility and cohesion, mood, communication, conflict, performance) have been investigated in natural analogs (e.g., Antarctic; Wood et al., 1999; Steel, 2001), space simulations (e.g., HUBES, Mars 105, SFINCSS; Gushin et al., 2001; Sandal, 2004; Nicolas et al., 2013), and isolated and confined laboratory settings (e.g., Emurian et al., 1984) dating back to at least the 1960s (e.g., Gunderson and Nelson, 1963; Altman and Haythorn, 1965; Gunderson and Ryman, 1967). This research suggests several dynamics unique to the LDSEM-analog settings.

As examples, while a meta-analysis of the traditional team literature suggests that the team cohesion and team performance relationship is generally small (Beal et al., 2003), team cohesion may be of particular significance when crewmembers live and work together and rely on one another for social support (Landon et al., 2015). Astronaut journals collected in the International Space Station (ISS) reveal a decreasing number of positive comments about team interaction over the course of a mission (Stuster, 2010). Further, problems associated with poor unit-level team cohesion such as subgrouping and isolation can occur, which have implications for conflict, information sharing, and team performance (Kanas, 1998; Kanas et al., 2009).

The psychological health of the crew is likely to be important for LDSEMs as crews will be living and working in an extreme environment for an extended duration. Communication between space crews and MC is thought to provide information about the crew's psychological health and the crew's psychological climate. Analysis of a space crew's communication with MC is the standard operating procedure of the psychological support group in Russian MC and is used to examine crews' emotional status and the communicators' coping strategies (Gushin et al., 2012, 2016). Among other things, research by Gushin et al., 1997, 2012 indicated that crews decreased the scope and content of their communication to outside personnel over time—a phenomenon called psychological closing.

Some crews have reported changes in mood over time. The third quarter phenomenon is the tendency for positive mood levels to decrease while negative mood levels and conflict increase after the midpoint of the mission (Bechtel and Berning, 1991; Steel, 2001; Dion, 2004; Kanas, 2004; Wang et al., 2014). Though mood is typically measured in LDSEM-analog research as an individual-level variable, researchers sometimes use the team mean of individual-level mood scores to represent team mood. Team mood is important because it contributes to team emotion, which is defined as a team's affective state that arises from bottom-up components such as affective composition, and top-down components such as affective context (Kelly and Barsade, 2001). Team emotion starts with individual-level moods and emotions and is then shared with the team either implicitly through emotional contagion or explicitly through means such as affect management. Environmental context such as lighting and physical layout can affect moods (see Kelly and Barsade, 2001). Thus, a better understanding of how team mood changes over time is necessary, especially given the extreme conditions expected for LDSEMs, such as living in a small transit vehicle with no natural light. The aforementioned evidence on team cohesion, communication, and mood are examples of findings that may be unique to the LDSEM context; this underscores the importance of examining team phenomena in LDSEM-analog environments.

While a body of research examines teams in analog environments, to date, it has not been quantitatively summarized. A quantitative summary of the analog team research is important for several reasons. First, it summarizes what we know about teams in LDSEM analog environments, given the available data. Specifically, it can provide insights into how team dynamics may unfold over time for LDSEM teams, and be used to benchmark typical and atypical team dynamics in the LDSEM environment. It also can identify potential threats to LDSEM team dynamics and performance. Second, it can help guide future research in analog environments by identifying what areas are in need of more research, new areas for research, and strategies that aid with knowledge accumulation over time. Guidance for future research is particularly important given the expense and time required to collect analog research.

### Objectives and Research Questions

The primary purpose of our research was to provide an overall picture of the available data on team dynamics and performance in LDSEM-analog environments. To do this, we systematically reviewed quantitative research conducted on teams in LDSEM-analog environments. We answer two primary questions with our systematic review: (1) which factors have been examined with quantitative research, and (2) what do these studies reveal about team dynamics in LDSEM-analog environments?

## Methods

### Study Design and Inclusion Criteria

Typically, meta-analysis is preferred for integrating estimates of the same relationship of interest across studies; it allows us to generate cumulative knowledge about a set of studies. The benefits of meta-analysis over narrative reviews have been widely noted (see Glass, 1976; Schmidt and Hunter, 2015). Early in our review process, however, we suspected that most studies conducted on teams in analog environments would not lend themselves to traditional meta-analysis. Frequentist meta-analytic techniques can be inappropriate when a limited number of studies have examined a particular relationship or when sample sizes or data do not permit the calculation of an effect size, for example, when data are only reported for a single team. Further, a review of the analog research at the individual-level determined that traditional meta-analytic techniques were inappropriate (e.g., Shea et al., 2011). Given this, our general approach (e.g., search strategies, coding) was consistent with best practices in meta-analysis in organizational psychology (e.g., Schmidt and Hunter, 2015); however, we retained a broader set of studies and ultimately used alternative analytic approaches to summarizing the data. Our reporting is consistent with the PRISMA guidelines (Moher et al., 2009) to the extent that they apply to non-medical systematic reviews.

We sought to be as inclusive as possible while also striving to ensure that the data were relevant to understanding team dynamics in an LDSEM environment. We applied three general inclusion criteria. First, we retained sources that reported quantitative data from teams in LDSEM-analog environments, however, we excluded descriptive case studies and narrative reviews. Second, we identified and included only team-level data (as opposed to individual-level data). We excluded articles that reported individual-level data that were not tied to a particular team (e.g., Bartone et al., 2002), or that were tied to a large polar station (>40 people) but not to a team or a small station (e.g., Doll and Gunderson, 1971; Palinkas et al., 1989). Third, we included research in which members of the focal team (e.g., the “crew” analog) live and work together for a period. We provide more detail on this decision next.

Defining an LDSEM-analog environment has challenges because a particular extreme environment (e.g., Antarctic winter-overs) may only share some of the same characteristics expected of LDSEM. All analogs are imperfect approximations of LDSEM, and researchers must weigh the importance of different features of the context in understanding the phenomena of interest. Because of this, we broadly defined LDSEM-analog research as research in which members of the focal team (e.g., the “crew” analog) live and work together for a period. We included military teams when they were expressly described as intact teams (e.g., combat teams; Ko, 2005; Lim and Klein, 2006) even if the research did not explicitly mention that the unit lived together. We did not include military or firefighter training exercises when it was unclear whether the team lived together either while at training or while not at training (e.g., Oser et al., 1989; Hirschfeld and Bernerth, 2008). We excluded sources that included data on children (e.g., Tyerman and Spencer, 1983). We coded features of the analog environment and sample characteristics as moderators, rather than excluding studies based on specific features of the analog (e.g., mission length, autonomy). We chose this approach so that we could make comparisons across different conditions (e.g., in isolated and confined setting, non-isolated confined settings; how phenomena change over time), as opposed to designating arbitrary cutoffs related to fidelity. It is important to note, however, that our decision criteria led to the inclusion of some missions in which teams lived and worked in an isolated and confined setting for shorter-durations (e.g., 6 and 10 days). We retained these in order to be able observe any potential changes over time, but note that they have lower fidelity in regards to duration.

### Search Strategy

We used a comprehensive search strategy to obtain quantitative research on teams in LDSEM-analog environments. Our efforts included: (1) searches of 13 databases that ranged from general databases such as Google Scholar and EBSCOhost, specialized databases such as the Military and Government Collection and space agency databases and technical report repositories (e.g., NASA, ESA, JAXA); (2) searches of specific journals such as Acta Astronautica, Aerospace Medicine and Human Performance, Human Factors; (3) contacting 29 researchers that we identified through the NASA taskbook, our project contact at NASA, or because they frequently publish in the area (e.g., Vinokhodova, Leon); (4) posts to listservs (e.g., Science of Team Science, INGRoup, relevant Academy of Management area listservs); and (5) a review of reference lists of key articles, including those from which we were able to obtain an effect size (e.g., Gunderson and Ryman, 1967; Emurian and Brady, 1984), reviews of similar domains (e.g., Schmidt, 2015), and recent technical reports on team research funded by NASA (e.g., Bell et al., 2015b; Burke and Feitosa, 2015; DeChurch and Mesmer-Magnus, 2015; Gibson et al., 2015; Smith-Jentsch, 2015). The search process included research published until November 2016. Researchers were contacted in May 2015.

### Data Sources, Studies Sections, and Data Extraction

In total, we identified approximately 309 sources (e.g., books, technical reports, dissertations, journal articles, and conference papers) for possible inclusion. To better understand the nature of the available data, we sorted the 309 sources into three categories: (1) sources that included quantitative data with a team-level sample size of 5 or greater, for which a team-level effect size between a predictor and criteria related to team functioning could be generated; (2) sources that included quantitative data on fewer than 5 teams or only data for one variable over time; and (3) sources that did not provide relevant data for our quantitative review. Sources in the third category were excluded from further review. The decision to exclude an article was agreed upon by at least two members of the research team. Seventy-two sources were retained for inclusion and coded for fidelity characteristics and other moderators, and the quantitative data on team dynamics. Of these, 11 different sources (e.g., journal articles, technical reports) provided enough information to calculate effect sizes representing the relationship between a predictor and a criterion related to team functioning, and 61 different sources reported quantitative data on team dynamics over time in LDSEM-analog environments but did not include enough data to calculate an effect size.

To extract data, two coding forms were created: one for coding effect sizes and one for coding data (e.g., means and standard deviations) related to team dynamics over time. When a source reported data on 5 or more teams and a predictor and team outcome relationships, we coded or calculated an effect size, either *r* or Spearman's *r*. For sources with a team sample size <5 we coded quantitative data such as means (or another team-level representation) and within-team standard deviation, when available, for team dynamics across time. We included data that were presented numerically as well as those presented in figures, except when the approximate value reported in the figure could not be reasonably estimated (e.g., due to ambiguities in labeling of the axis).

Coding forms were similar in that both captured characteristics of the source, the sample, fidelity characteristics, and information about the predictor and/or criteria. In addition, a codebook with definitions of the variables and descriptions for the different categories for each variable was developed. We coded fidelity characteristics when they were described or could be reasonably assumed by two independent coders, given the descriptions provided in the sources. We used the Internet to locate information about specific simulations or Antarctic stations to complete missing fidelity information, where possible.

We coded study design as: (a) descriptive, (b) correlational, (c) quasi-experimental, and (d) experimental. We coded the degree of similarity between the sources' samples and the anticipated characteristics of LDSEM crews in terms of demographic differences (e.g., gender, national background). We coded the fidelity of the team to the characteristics expected for LDSEM crews. Studies were coded as occurring in dangerous environments when the setting had features that required individuals to use special equipment (e.g., winter-overs in Antarctic) or posed an imminent threat (e.g., polar bear threat). Studies were coded as isolated when team members were limited in physical interaction with outside parties for a substantial period of time during the study, and confined when they primarily operated in a highly restricted space. For example, winter-overs in small Antarctic stations or space simulations were coded as an isolated and confined environment. Autonomy was coded as high, moderate, low, or not reported. Many studies did not describe the level of autonomy in detail and were coded as “not reported.” Mission length was coded as the total of number of days in the team's life span. Ongoing teams such as firehouses (e.g., Kniffin et al., 2015) were coded to the max of the distribution (e.g., 730 days). We also coded crew size, workload amount and variability, how the crew communicated with those outside of the focal crew (e.g., mission control) and whether there was a time delay in the communication.

### Coder Training and Agreement

The second and third authors served as coders for this study. The primary author trained the coders on the coding scheme described in the previous section. Coders first received a coding sheet and a codebook that provided descriptive information about each category of variables. All three authors then used the codebook and coding sheet to independently code three articles. The three authors met to discuss the coding, observe areas of agreement and disagreement, and make modifications to the coding sheet and codebook. Next, all three authors recoded the initial set of articles to help establish a frame of reference that incorporated the modifications made to the coding documents. Disagreements about the coding were resolved during a follow-up meeting using a consensus approach. After the second round of coding, a common set of 5 articles was coded to determine the efficacy of the coding process and to establish decision rules. When there was little disagreement (i.e., <3 disagreements across the variables coded in the studies), two coders coded the remaining articles. A randomly sampled common set of coded articles indicated that initial agreement, prior to the consensus meeting between coders, was relatively high (mean agreement of 87% on the variables that were coded). Discrepancies between the two coders were discussed and agreement was reached using a consensus approach. When consensus could not be reached with certainty between the two coders, the coders met with the primary author to discuss how the characteristic in question should be coded. After the coding was completed, we inspected the data sets to better understand the nature of the data, to determine the appropriateness of meta-analysis for summarizing the data, and to determine the best way to summarize the available evidence.

### Analytical Strategy

Although we were able to locate a relatively large amount of data for our review, the small sample sizes in most studies (e.g., <5 teams) and the variety of relationships examined in the effect size studies, suggested the majority of the data did not lend themselves to traditional meta-analytic techniques. Thus, we used the following approaches. First, when the team factor and team outcome relationship could be represented using an effect size, we calculated a weighted average of the effect size from the local (analog) population and the relevant meta-analytic estimate from the traditional teams literature as a minimum-variance estimate. We used this approach as a means of balancing the precision that meta-analysis can provide in estimating a relationship across multiple settings with the high uncertainty (especially due to small sample sizes, etc.) but localness that a specific effect size generated in an LDSEM-analog environment can provide. We also calculated the average inaccuracy of the estimates and used these to create 95% credible intervals to quantify the uncertainty of the estimates.

We used equations 1, 2, 3, and A12 from Newman et al. (2007) in forming our weighted averages. We used estimates from meta-analyses in the extant literature (e.g., Beal et al., 2003; LePine et al., 2008; Bell et al., 2011) to inform the prior probability distribution. We only generated an estimated distribution of the true population local validity when there was a relevant meta-analytic effect reported in the extant literature that could inform our prior distribution. This limited the number of relationships we estimated and narrowed the effects to team performance as the outcome. Further, even with performance as the outcome, there were a number of relationships for which we could not locate relevant meta-analyses; the relationships between leader-member exchange—the idea that leaders have relationships with their followers that vary in quality (Graen and Uhl-Bien, 1995)—and team performance, and between personality characteristics (e.g., conscientiousness) of the leader and team performance are examples. We also did not locate relevant meta-analyses for many of the personality and needs variables examined by Gunderson and Ryman (1967), such as wanted affection and nurturance personality. Finally, there were two estimates from military teams [e.g., shared mental models from Lim and Klein (2006) and collectivism from Ko (2005)] that were already included in meta-analyses that would have been used in the calculation of the weighted averages [(DeChurch and Mesmer-Magnus, 2010); and Bell (2007) respectively]; we did not estimate local validity of these two estimates. We corrected the observed correlations in a given analog study for unreliability of the predictor and criterion in order to match the corrections used in meta-analyses that were used to inform our prior distribution. Although we would have preferred not to correct the local validity estimates for unreliability because of the small sample sizes on which they were based, the majority of the variances used to inform the prior distributions were corrected for unreliability. Newman et al. (2007) indicate the importance of ensuring that the prior and local effects have the same corrections. When reliability was not reported, we used the closest estimation of reliability from the most similar research in our data set. When the correction resulted in an estimate >1, we did not compute a weighted average. This is because the weighted averages approach relied on the z transformation, which for values over 1 is undefined. Values exceeded 1 for correlations from Gunderson and Nelson (1963) and Gunderson and Ryman (1967), which were based on the same source data (e.g., self-report cooperation and performance) and exceeded 0.90 prior to correction.

Second, when the number of teams included in the study was too few to generate an effect size, and when data across studies were comparable, we descriptively summarized the data on team dynamics over time via a series of figures. We plotted team dynamics over time when data were comparable (e.g., similar scales, similar response formats), and reported for at least three different teams across at least two different data sources (e.g., articles, conference presentations). We plotted team dynamics over time in terms of mission days and over relative time. Relative time was calculated as the mission day divided by the total mission length. Relative mission time was examined given that some effects for factors such as team cohesion and conflict, are thought to different because of the point of the team in the lifespan (e.g., third quarter phenomenon) rather than the mission day itself.

## Results

### Flow Diagram of the Studies Retrieved for the Review

**Figure 1:**
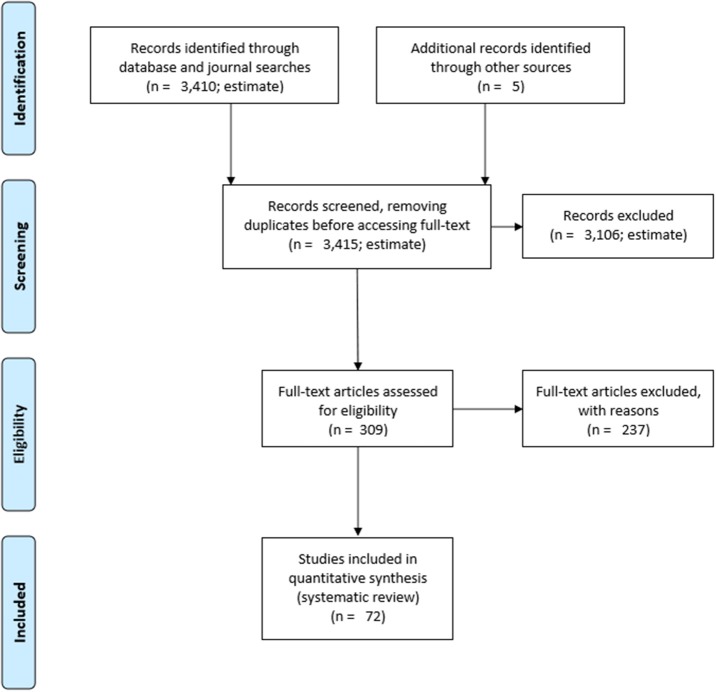
PRISMA 2009 Flow diagram. Moher et al. (2009).

Figure 1 depicts the flow diagram of the studies retrieved for review.

### Study Selection and Characteristics

Eleven sources (e.g., journal articles, technical reports) provided enough data (team *n* ≥ 5) to generate 253 team-level effect sizes that represent a team factor (e.g., team cohesion) and team outcome (e.g., team performance) relationship. We refer to this as our effect size data set. Sixty-one sources included data on team functioning from fewer than 5 teams; from these sources we were able to glean 1,150 data instances (i.e., data collected on one or more variable at a particular time point) to benchmark team dynamics in LDSEM-analog environments over time. We refer to this as our benchmarking data set. We provide a summary of the fidelity characteristics of our samples in Supplementary Table 1.

### Synthesized Findings

Our first research question asked: what factors related to team dynamics has quantitative research examined in analog environments? In the effect size data set, the majority of effects (i.e., 102 effects across 9 studies) represented the relationship between a predictor and team performance. Forty-seven effects across 6 studies represented the relationship between a predictor and cohesion or compatibility, and the remaining effects represented a variety of outcomes that differed across studies. The specific predictor and criterion relationship examined varied across studies. Predictors included inputs, emergent states, and team process variables (see Marks et al., 2001), personality (e.g., Gunderson and Ryman, 1967), values, leader-member exchange, and team-member exchange (e.g., Ko, 2005), compatibility and cohesion (e.g., Gunderson and Nelson, 1963), mental models (e.g., Lim and Klein, 2006), conflict (e.g., Seymour, 1970), leadership (e.g., Lim and Ployhart, 2004), ability, experience, mood, exploratory search, and planning (e.g., Knight, 2015). Outcome variables included performance effects (e.g., accomplishment, accuracy, time to completion, efficiency, and quality), emergent states, team processes, and other team dynamics such as cohesion, team mood, egalitarian atmosphere, viability, team-member exchange, leader-member exchange, exploratory search, and cooperation. The data were largely dependent (i.e., the 253 effects came from only 11 different sources), and a variety of predictor and outcome relationships were examined. Only the relationship between measures of cohesion (e.g., compatibility, spending time together) and team performance was examined in more than 3 independent samples (*k* = 6).

In the benchmarking data set, team factors included emergent states, team processes, outcomes, and additional team dynamics markers. For example, emergent states included team cohesion (e.g., Allison et al., 1991; Vinokhodova et al., 2012), and team processes included conflict and interpersonal relations (e.g., Leon et al., 2004; Šolcová et al., 2014). Outcomes included performance (e.g., Emurian and Brady, 1984) and more subjective outcomes such as satisfaction (e.g., Bhargava et al., 2000; Leon et al., 2004). Finally, other dynamics markers, such as team mood (e.g., Kahn and Leon, 2000; Steel, 2001; Bishop et al., 2010), were commonly reported in analog studies. A full list of all team factors examined for the effect size and benchmarking data sets is available in Supplementary Table 2.

Our second research question asked what quantitative research reveals about team functioning in LDSEM-analog environments. We discuss the results of the weighted averages approach, and descriptive figures benchmarking team dynamics next.

### Weighted Averages Approach

**Figure 2:**
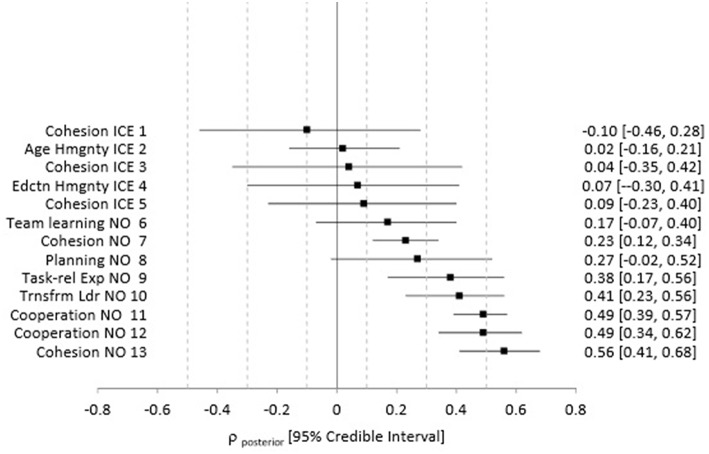
Estimated distributions for the predictor and team performance relationship in analog environments. ICE = analog team was in an ICE environment (e.g., Winter-overs in Antartica) NO = team was living and working together but not in an ICE. Hmgnt = Homogenity. Task-rel Exp = task-relevant experience. Trnsfrm Ldr = Transformational Leadership. The square represents the weighted average local validity population estimate (ρ_posterior_) and the bar represents the 95% credible interval. Specific estimates are provided in the right column as per ρ_posterior_ [95% credible interval]. The credible interval can be interpreted as follows: there is a 95% chance that the true population predictor and team performance relationship (ρ) is between the first number and the second number. Number in the left column indicates the analog data source. 1. Gunderson and Nelson (1963), Outcome = self-report team achievement, Antarctic stations; 2 and 4 from Gunderson and Ryman (1967), Outcome = team accomplishment, mixed sources, Antarctic stations; 3. Emurian and Brady (1984), outcome = performance on lab task; 10-day isolated lad experiment; 5. Nelson (1964), outcome = supervisor ratings of individual performance aggregated within station, Antarctic station; 6, 8, and 9 Knight (2015), outcome = team's time and number of obstacles completed in a final challenge task, military training; 7 and 11 Kniffin et al. (2015), outcome = supervisor rating of performance, firehouses; 10, 12, 13. Ko (2005), outcomes = team performance, mixed sources, special operations teams.

We used our weighted averages approach to provide the best possible estimate of the magnitude and direction of the relationships between team factors and team outcomes in the analog environments, given the available data. Figure 2 summarizes the weighted averages results, the credible intervals around the estimates, and displays the forest plot. Specific information about the local validity information obtained from LDSEM-analog studies, the meta-analytic effects that we used in the calculation of the weighted averages, and the estimated posterior distributions are provided in Supplementary Table 3. Local validity estimates include team performance with cohesion, age homogeneity, education homogeneity, team learning, planning, team task-relevant experience, cooperation, and transformational leadership.

First, we discuss the team cohesion and team performance relationships. Studies 1, 3, and 5 (as noted in Figure 2) were conducted on teams in isolated and confined environments (ICE); each of these studies measured team cohesion and team performance with different operationalizations. Estimates 7 and 13 reflect the team cohesion and team performance relationships for teams that are sometimes used as LDSEM-analogs but which are not isolated or confined for extended periods (non-ICE).

That data suggest that with 95% certainty, we cannot speak to the direction or size of the team cohesion and team performance relationship in ICE. For example, estimate 1 reflects the estimated results for the team cohesion and team performance relationship for data collected in Antarctic stations where team cohesion was operationalized as self-rated compatibility of station members, and team performance was operationalized as self-rated station achievement. The mean estimated validity is −0.10, and with 95% certainty, we estimate that the true population validity falls between −0.46 and 0.28. This is rather imprecise, as the prediction interval includes large, moderate, and small negative effects, no effect, and small and moderate positive effects. Conversely, with 95% certainty, we can describe the team cohesion and team performance relationship in the firehouses studied as positive and small to moderate (i.e., estimate 7), and in the special operations military teams studied, as positive and moderate to large (estimate 13).

Data for a few additional relationships other than team cohesion and team performance were also available. The age homogeneity and team performance (Figure 2, Estimate 2) and the educational level homogeneity and team performance relationships (Figure 2, Estimate 4) in an ICE (e.g., Antarctic station winter-over) were estimated with a large degree of imprecision; the prediction interval included positive, negative and no effect. Conversely, with 95% certainty, the true population effect between cooperation and team performance is estimated to be positive and large (Figure 2, Estimates 11, 12) for firehouses and special operations teams. Finally, with 95% certainty, the true population effect between transformational leadership and team performance for special operations teams, and the true population effect between team task-relevant expertise and team performance for military training teams are positive and exceed a small effect (Figure 2, Estimates 9, 10).

Taken together, there is a high degree of imprecision associated with estimates of the true predictor and team performance relationships from studies with teams in ICEs. Specifically, unlike most of the estimated relationships from teams in non-ICE, given the current data, if we retain a 95% level of certainty, we have limited to no understanding of the size or direction of the relationship of team cohesion and team performance observed in multiple ICE, age homogeneity and team performance in an ICE, and educational homogeneity and team performance in an ICE.

### Benchmarking Team Functioning Over Time

Next, we benchmarked team dynamics over time in studies with sample sizes too small to generate a between-team effect size, but for which data were comparable (e.g., similar measures, similar response formats) on at least three different teams from at least two different data sources (e.g., articles, conference presentations). With this requirement, we were able to generate figures on cohesion, efficiency, team conflict, communication with MC, and team mood.

### Team Cohesion

**Figure 3:**
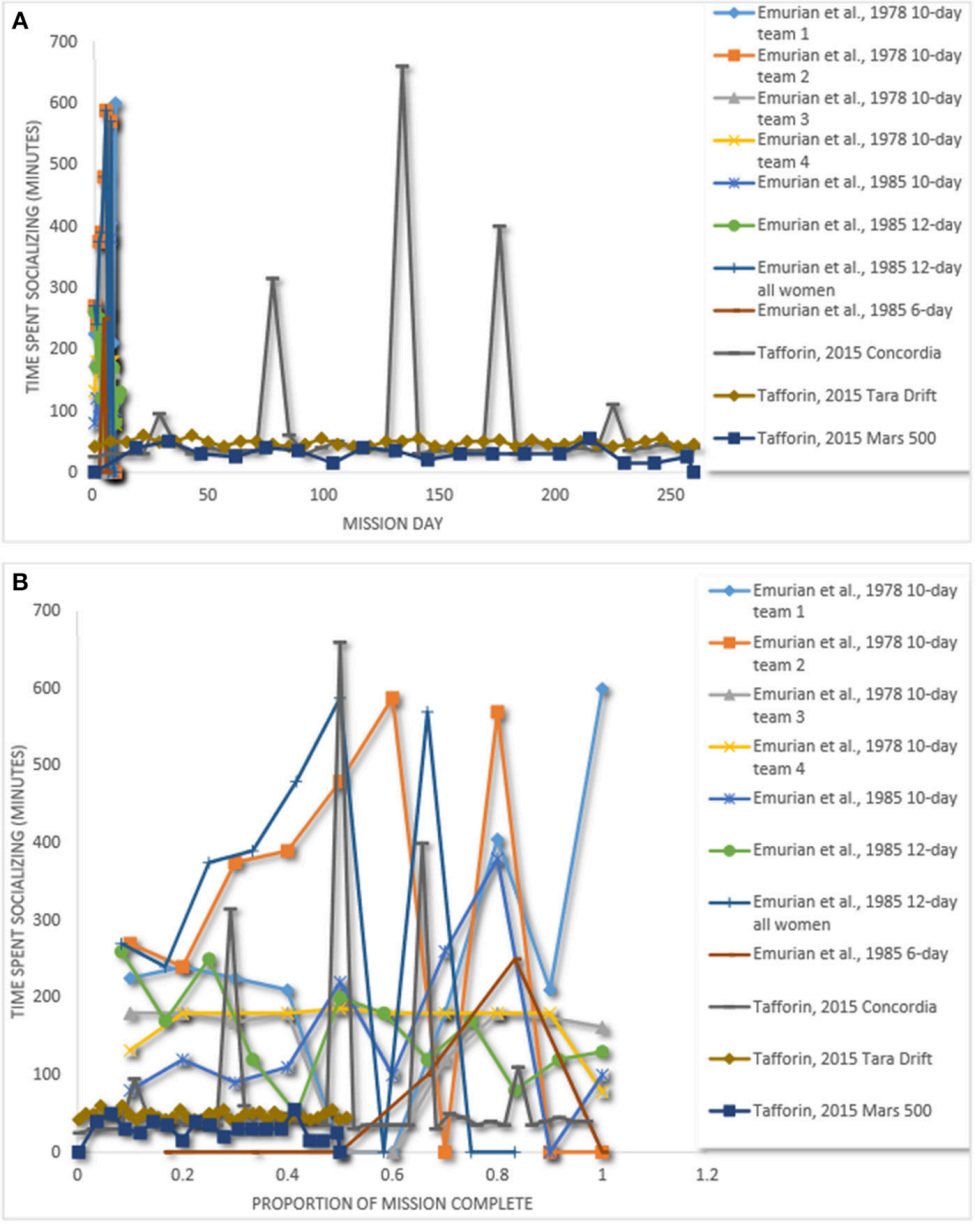
**(A)** Team cohesion over time. **(B)** Team cohesion over relative time. Emurian et al. (1978), Emurian et al. (1985), Tafforin (2015).

While we identified several studies with cohesion data reported over time from 5 or fewer teams, these data were collected using a variety of cohesion operationalizations making it difficult to directly aggregate and make for meaningful comparisons across settings. We were able to benchmark a subset of this data by identifying 3 sources with data from 11 teams spending time together (e.g., social activities, eating meals). We classified these activities as evidence as social cohesion. Figure 3A illustrates team cohesion across mission days. Figure 3B plots team cohesion over relative time (i.e., the mission day divided by the total mission length).

The data reported suggests some fluctuations in cohesion over time. However, two patterns are present. First, it appears team members spend more time together during shorter missions. The Concordia, Tara Drift, and Mars 500 missions lasted for 268, 507, and 520 days, respectively. In comparison to shorter missions [i.e., Emurian et al., 1978, 1985], which lasted for 6, 10 and 12 days, team members in longer simulations generally spent less social time together. There was one exception to this: time together increased sharply at certain points for a team at Concordia station. These instances could have been the result of significant events at the station during those periods (Tafforin et al., 2015). It is important to note that we included shorter-duration missions to avoid an arbitrary cut off and to observe changes over time. The stark contrast between shorter-duration and longer-duration missions on time spent together suggest limited usefulness of shorter-duration studies in understanding team cohesion for LDSEM.

### Team Performance

**Figure 4:**
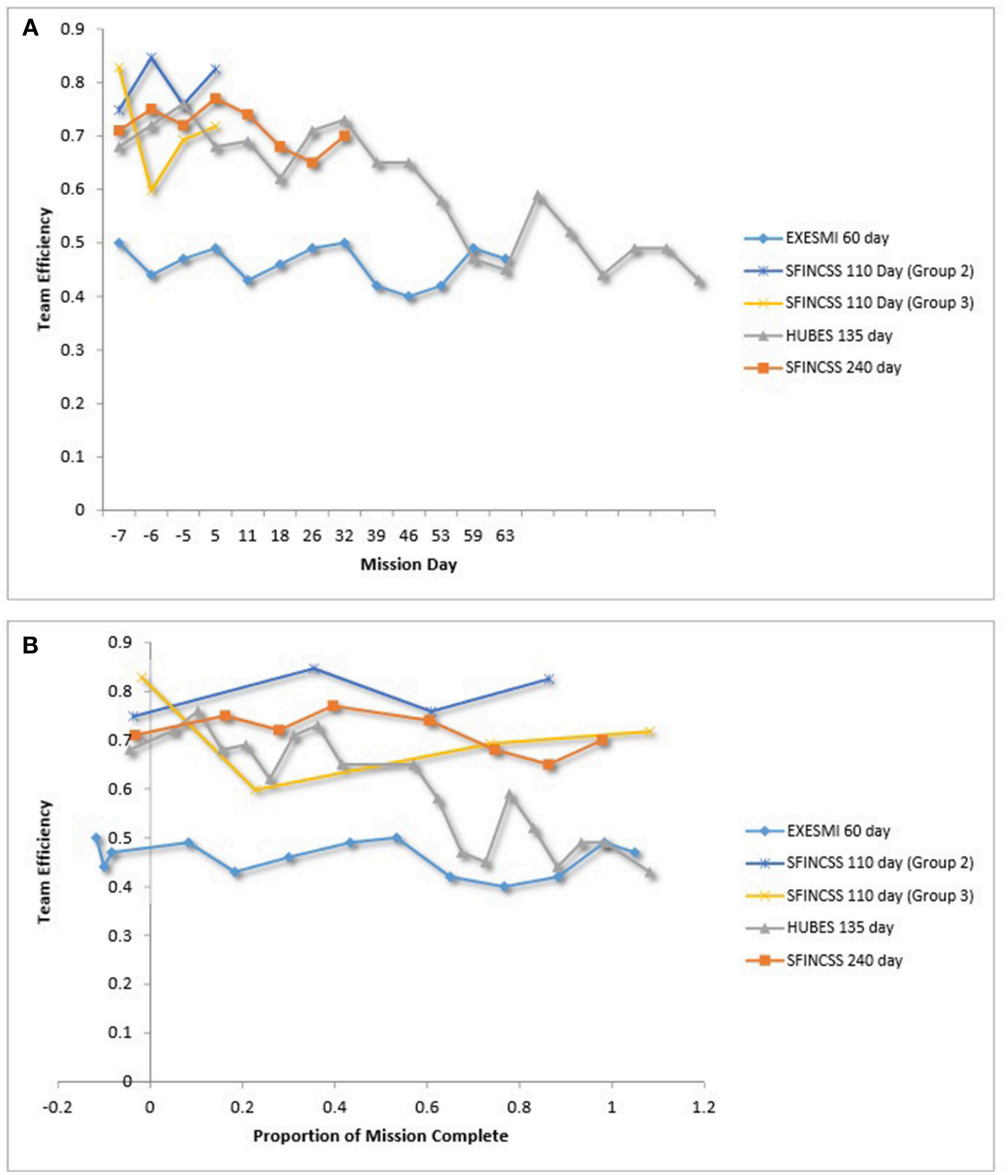
**(A)** Team efficiency over time. **(B)** Team efficiency over relative time. Le Scanff et al. (1997), Vinokhodova et al. (2001), Eskov (2011).

Homeostat was used to collect data on team performance across a number of space simulations (e.g., HUBES, SFINCSS). Homeostat is a computer task in which, under time pressure, a team solves tasks that require the coordinated action of the whole team (Eskov, 2011). A number of metrics can be assessed using Homeostat, including an efficiency metric (Csh) and leadership tactics. Figure 4A is a plot of team efficiency across mission days. Figure 4B is a plot of team efficiency over relative time.

The data suggest that three teams (i.e., a team in EXEMSI and two of the teams in SFINCSS simulations) were relatively consistent in terms of efficiency over time. The HUBES team decreased steadily in efficiency over time. One of the SFINCSS teams (Group 3) had a sharp decline in efficiency early in the simulation and then steadily increased during the remainder of the simulation.

Descriptive information on team dynamics in the HUBES and SFINCSS simulations implicate ineffective role structure and conflict as possible triggers of the performance decrements of HUBES and SFINCSS–Group 3. Specifically, in addition to measures of efficiency, the Homeostat also collects information on leadership tactics by individual team members as a means of understanding the leadership structure used while completing the task. For SFINCSS group 3, Vinokhodova et al. (2002) indicated that the data did not suggest that a role distribution structure had sufficiently developed. Further, the SFINCSS simulation also included a New Year's Eve incident between a member of another group and a woman in Group 3 of the simulation, which led to tension between crews (Sandal, 2004). The sharp decrease in effectiveness in the SFINCSS Group 3 depicted in Figure 4A also happened around this time. For HUBES, Sandal (2001) reports that there was evidence of an unstable crew structure; specifically, the commander's leadership was challenged during the first 8 to 10 weeks of the mission. Further, crew relations in the simulation were marked by interpersonal tension and alienation of one crew member during later parts of the experiment. Taken together, this may suggest ineffective role structure, conflict, and alienation as possible threats to team efficiency.

### Team Conflict

**Figure 5:**
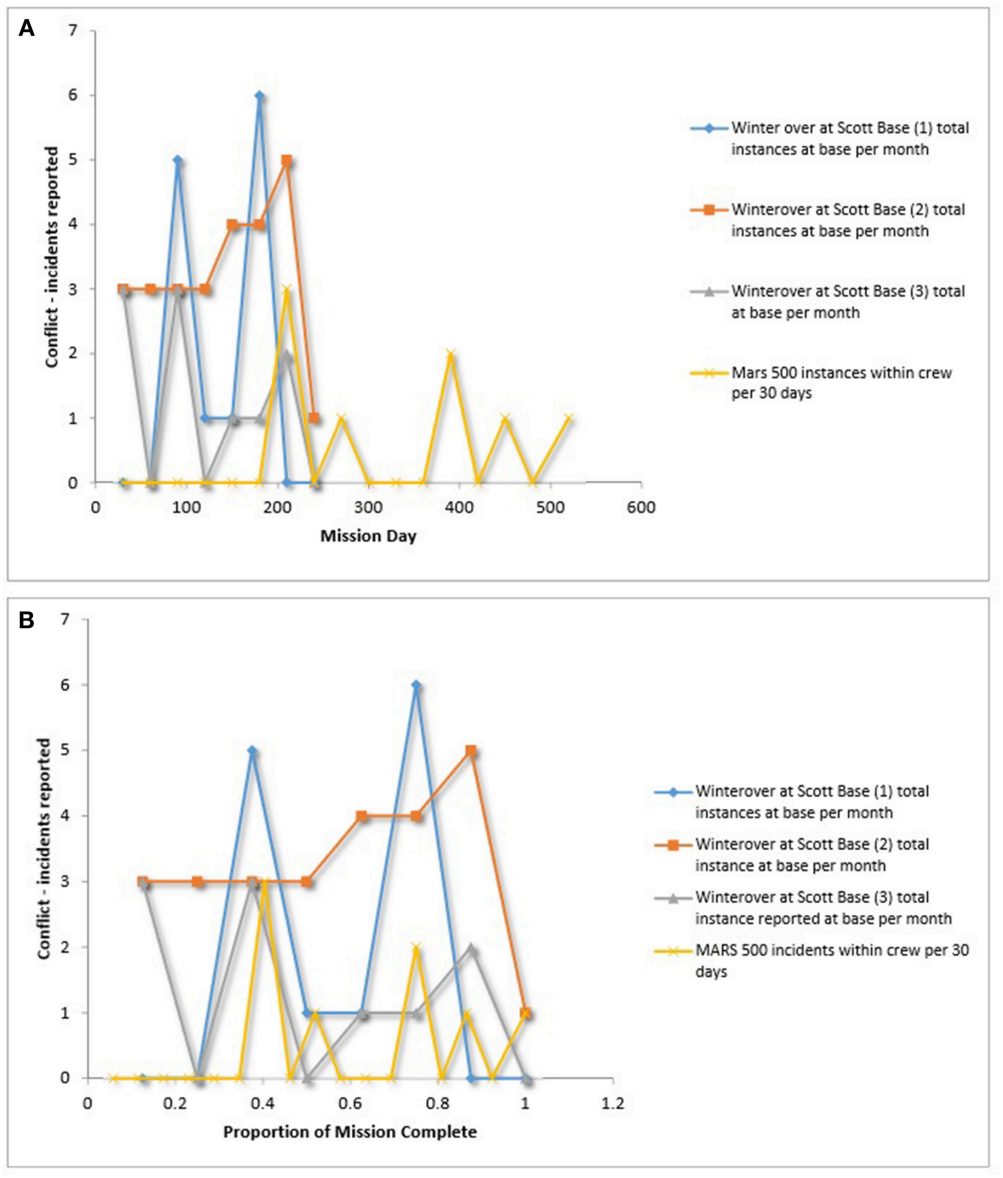
**(A)** Team conflict over time. **(B)** Team conflict over relative time. Steel (2001), Basner et al. (2014).

A few sources (*k* = 4) reported conflict scores over time for 8 different teams using 2 types of conflict metrics (e.g., total number of conflicts reported, Likert scale). Figures 5A,B summarize data that were comparable across multiple teams from different analog environments for the total number of conflicts reported within crews. Data do not show a consistent trend across teams. Some teams are more variable than others in the number of conflict incidents per month, while others are more stable. Some teams report conflict early on, while others do not. By 40% of the mission completion (with this data the equivalent of at least 90 days) all teams had reported a least one instance of conflict. No team had more than six instances of conflict per month with a given target (i.e., the crew or MC).

### Communication With Mission Control (MC)

**Figure 6:**
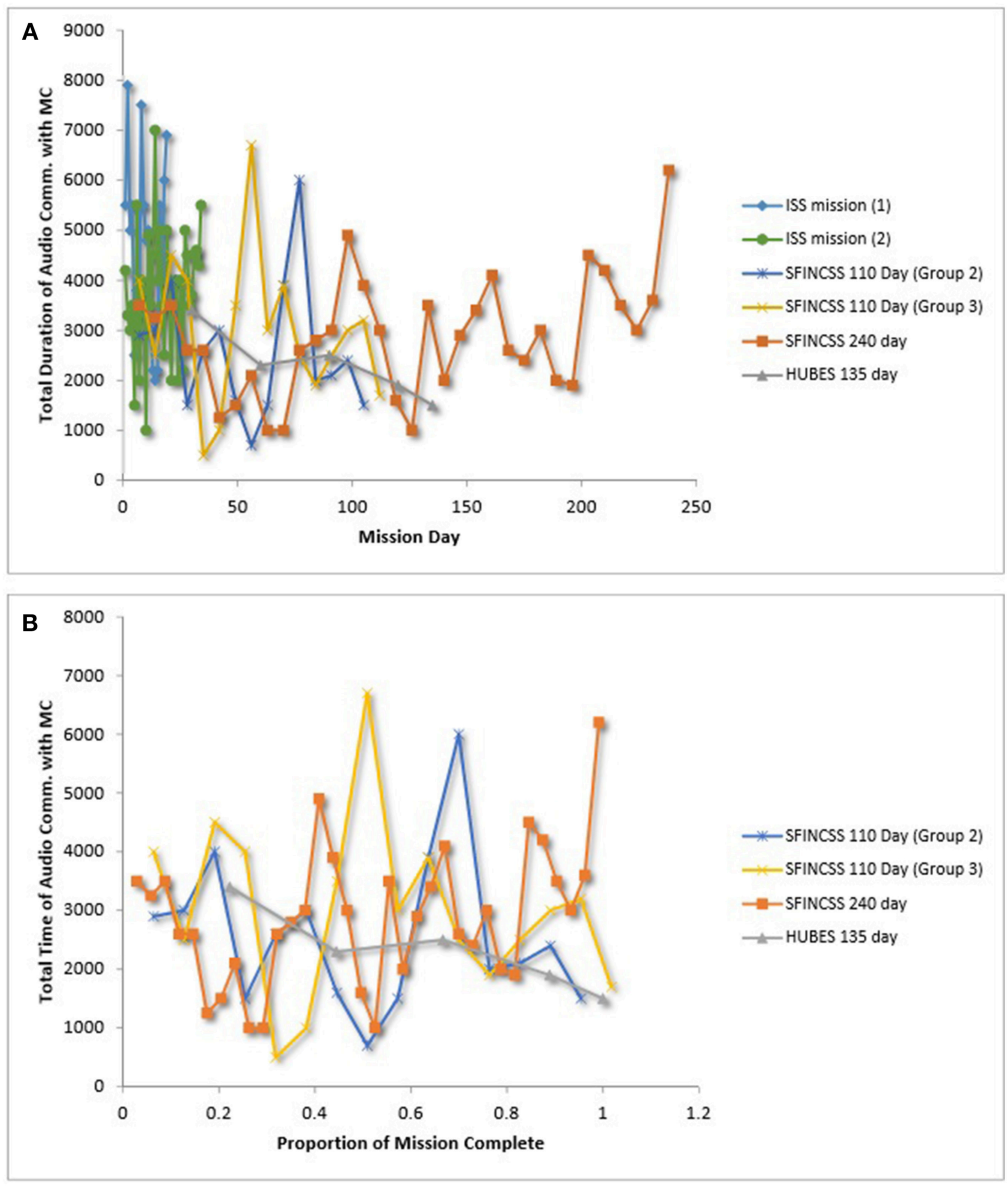
**(A)** Team communication with mission control over time. **(B)** Team communication with mission control over relative time. Gushin et al. (1997), Gushin et al. (2001), Gushin and Yusupova (2003).

**Figure 7:**
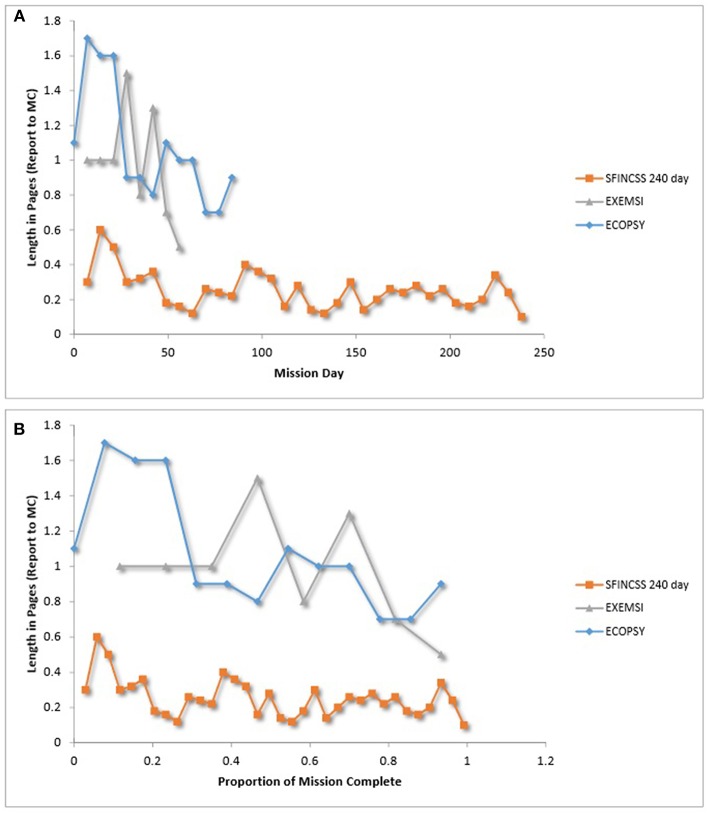
**(A)** Commander report length over time. **(B)** Commander report length over relative time. Gushin et al. (1996b); Gushin et al. (1997), Gushin et al. (2001).

Gushin et al. have examined crew communication with MC in several studies (e.g., Gushin et al., 1997, 2001; Gushin and Yusupova, 2003) and have reported comparable data, which allowed us to plot the total duration of crew–MC audio-communication sessions (in seconds) over time (see Figures 6A,B), as well the average report length per week of the commander's end-of-day report to MC (see Figures 7A,B). For the SFINCSS, HUBES, and ECOPSY simulations, audio communication paralleled the standards of Mir in that 30 min were made available for audio communication every 90 min in the daily schedule but use of the time was not required. At the end of each day, the commander submitted a written report to MC on mission status and fulfillment of the daily schedule (Gushin et al., 1997, 2001). Data in Gushin and Yusupova (2003) was collected by researchers listening to crew-MC communication once a week (for ISS mission 1) and twice a week (for ISS mission 2).

As depicted in Figures 6A,B, patterns of average audio-communication length between the commander and MC were inconsistent across teams. It is interesting to note, that the HUBES crew that had decreasing efficiency over time (Figure 4A) also had shorter audio communication with MC over time (Figures 6A,B). As depicted in Figures 7A,B, average mission report length to MC per week decreased over the course of the mission in SFINCSS, EXEMSI, and ECOPSY. Gushin et al. (2012) describe this as the closing of a communication channel, or psychological closing. Psychological closing can include a decrease of the communication volume throughout isolation, decrease in the issues discussed, and preference for communication partners.

It should be noted that there is a wealth of specific details (e.g., negative statements, jokes) that can be gleaned and assessed via content analysis of within- and between-group communications. Our figures here only reflect report length and total time for audio communication, which were reported in the same format across multiple teams. We refer the interested reader to Gushin et al. (2012) and Tafforin (2015) for more detail on the range of communication parameters that have been examined.

### Team Mood

**Figure 8:**
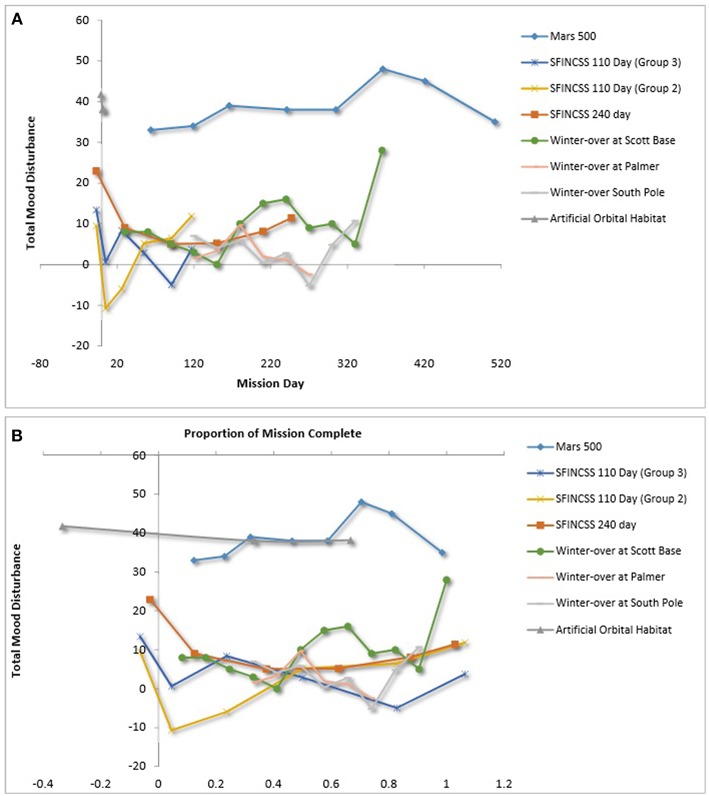
**(A)** Team mood disturbance over relative time. **(B)** Team mood disturbance over relative time. Palinkas and Houseal (2000), Steel (2001), Vinokhodova et al. (2002), Sandal (2004), Wang et al. (2014).

Multiple studies reported the affect of team members using Profile of Mood States (POMS; Shacham, 1983; Curran et al., 1995). POMS captures individuals' mood via self-report ratings on six dimensions using a 5-point Likert scale. The dimensions are tension-anxiety, depression-dejection, anger-hostility, fatigue-inertia, confusion-bewilderment, and vigor-activity. To arrive at an overall total mood disturbance score, the first five subscales listed are summed and then the vigor-activity subscale is subtracted. Team mood is captured with the average total mood disturbance across the team. Figures 8A,B show team mood over time and team mood over relative time, respectively. Figure 8A shows that the MARS 500 crew reported elevated total mood disturbance compared with teams in other LDSEM-analog environments, although it should be noted that the scaling reported for Scott Base was 0 to 4 instead of 1 to 5 as in the other simulations. Thus, the winter-over at Scott Base may have ratings more similar to Mars 500. Both studies that included teams in ICE for a year or more (e.g., Mars 500, an Antarctic winter-over) showed a spike in team total mood disturbance around the 1-year mark, and this was confirmed in the text of the studies reporting the data (e.g., Steel, 2001; Wang et al., 2014). Figure 8B, which shows total mood disturbance over time relative to the proportion of the mission complete, does not support a clear third-quarter phenomenon at the team level.

**Figure 9:**
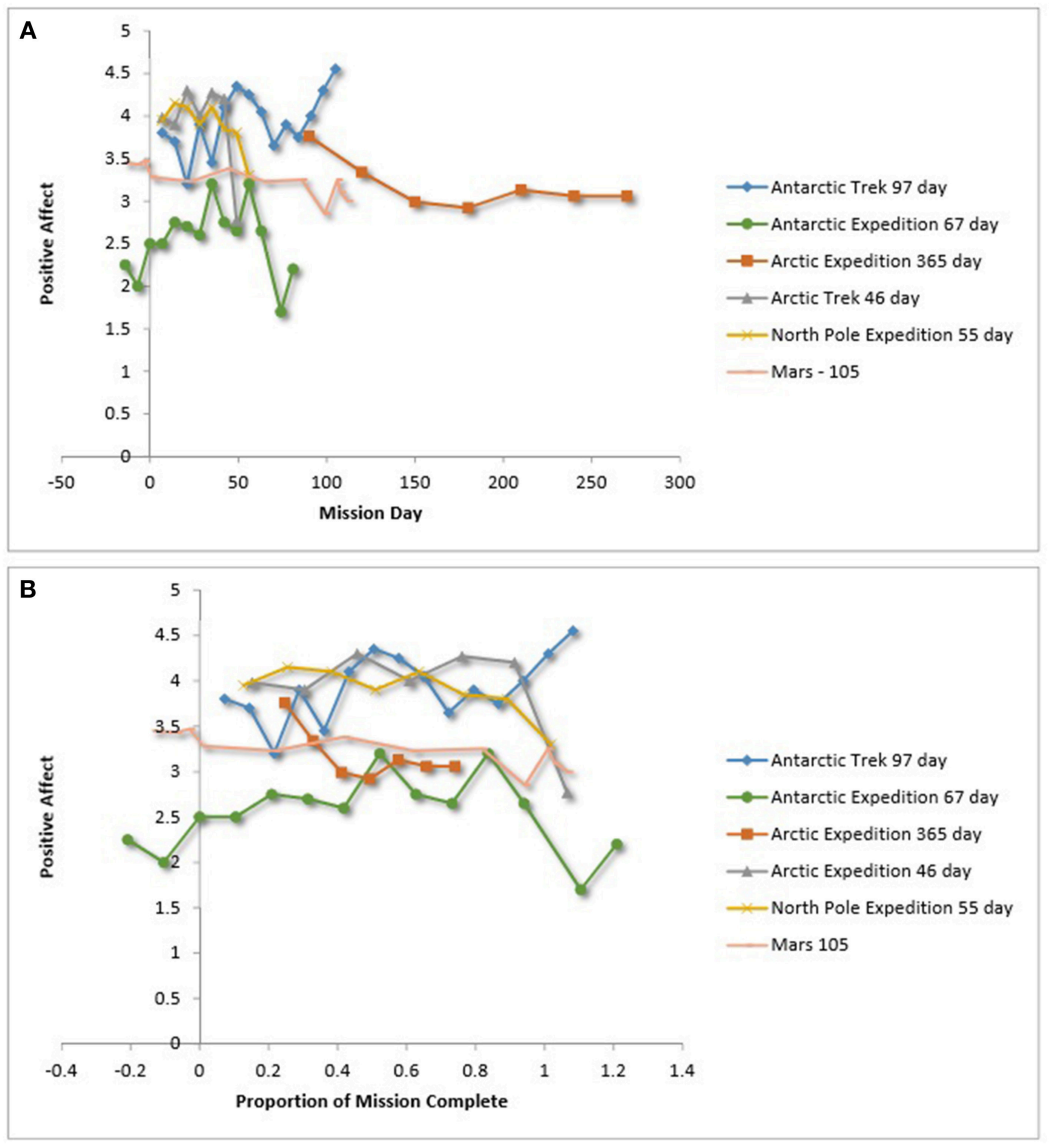
**(A)** Team positive affect over time. **(B)** Team positive affect over relative time. Kahn and Leon (2000), Steel (2001), Atlis et al. (2004), Leon et al. (2004), Leon et al. (2011), Nicolas et al. (2013). Binsted (2015) provided unpublished data that may be later published. Because of the level of granularity of these figures, the data are not displayed.

**Figure 10:**
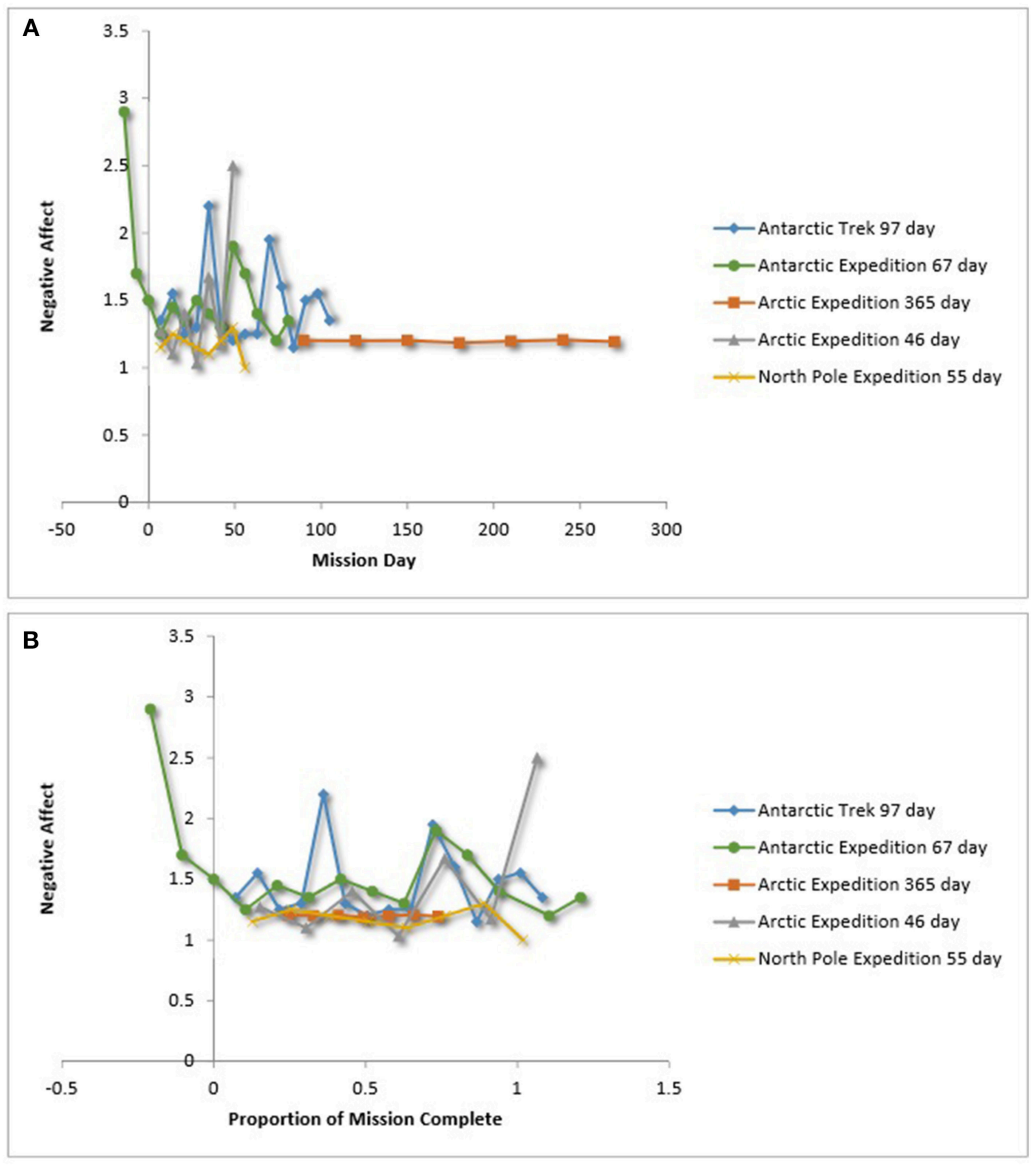
**(A)** Team negative affect over time. **(B)** Team negative affect over time. Kahn and Leon (2000), Steel (2001), Atlis et al. (2004), Leon et al. (2004), Leon et al. (2011), Nicolas et al. (2013). Binsted (2015) provided unpublished data that may be later published. Because of the level of granularity of these figures, the data are not displayed.

Team mood also has been operationalized in LDSEM-analog environments as the team mean of self-report ratings on the positive and negative mood components of the Positive and Negative Affect Schedule (PANAS; Watson et al., 1988, see Leon et al., 2004, 2011, for examples). Figures 9A,B,10A,B, show the relationship between affect operationalized as the team mean PANAS scores over time. Figures 9A, 10A show team positive affect over time and relative time. Figures 9B, 10B show team negative affect over time and relative time. For team negative affect over relative time, three of seven LDSEM-analog teams show an increased negative affect during the third quarter.

### Risk of Bias

There are two key risks of bias in our systematic review. First, publication bias may be a problem, especially given the small sample sizes associated with analog research. More extreme findings are more likely to be published. Small sample sizes compound the issue because the extreme findings are less likely to replicate. Given this, we made a focused effort to obtain unpublished research. Second, there were two potential biases associated with our weighted analyses approach. Some of the effect sizes used in our weighted averages approaches were based on very small sample sizes, which may influence the normality of the local validity distribution. We based our weighted averages approach on Newman et al. (2007) local validity Bayesian estimation approach. However, the local validity Bayesian approach is only regarded as Bayesian when the distribution of the local estimate is normally distributed. Because it is not possible for us to test this assumption without access to raw data, we referred to our approach as taking a weighted average.

Further, due to the limited amount of data in different analog conditions, we were unable to estimate potential bias due to certain moderators such as whether the analog study was conducted in an ICE or non-ICE environment. However, Newman et al. (2007) indicates that the accuracy of their local validity Bayesian estimation approach holds true even in the presence of true moderators (e.g., teams that perform in ICE environments, for example, where the ICE/non-ICE context moderates the observed predictor and outcome relationship). Even so, we acknowledge that because we cannot assess or model the bias that may be present due to combining a local effect size from an ICE environment with a meta-analytic effect from non-ICE environments, we are trading an unknown amount of bias to generate a minimum variance estimate. If raw data were available, it would be better to do a full Bayesian analysis that takes into account sampling variability at the local level, as well as any bias in using a meta-analytic estimate based on the broader team literature as the prior distribution. Given the limitations of available data, however, we believe our weighted averages approach provides the best estimate of the team predictor and outcome relationships in the specific LDSEM-analog environment. Further, given the limitations of the data from sources, which had fewer than 5 teams, we believe our descriptive figures best represent the data.

## Discussion

LDSEMs such as human missions to Mars are of increasing interest to NASA, space agencies, and private sector organizations. Conducting research in analog environments provides a means for understanding team dynamics for a potential LDSEM mission as well as other teams operating in similar ICE environments (e.g., oil drilling teams). Analog research on team dynamics has a long history dating back to at least the 1960's, thus it is important for researchers and agencies to learn from the past analog research to inform future analog research and prepare for future space exploration. The primary goal of this research was to summarize the existing quantitative evidence on team dynamics in LDSEM-analog environments.

### Summary of Main Findings

Our study has three key takeaways. First, there is an extensive research base on teams in LDSEM-analog environments. We were able to locate 72 different sources reporting quantitative research. Although there are quite a few studies that have examined teams in LDSEM-analog environments, the major of the studies had too small of a sample size to generate a between team effect size. Inconsistency in how the same construct was measured across studies further limited the ability to make comparisons across studies. Second, team dynamics are dependent on specific aspects of the context. For example, the team cohesion and team performance relationship was positive and strong for teams that lived and worked together but not in isolation and confinement (e.g., special forces teams), while little could be said about the relationship between team cohesion and team performance for teams in isolation and confined environments—an important aspect of LDSEM. Further, team dynamics varied greatly over time, underscoring the importance of temporal considerations and fidelity in analog environments. Third, we were able to document and provide interesting insights into how team dynamics unfold over time. These benchmarking figures provide insights into how team dynamics may unfold over time for LDSEM teams, benchmark typical and atypical team dynamics in the LDSEM, and identify potential threats to LDSEM team dynamics and performance. More detail on specific findings is provided next.

Results from our weighted averages approach suggest that the team cohesion and team performance relationship may be operating differently in isolated and confined environments (e.g., Antarctic stations, laboratory research with ICE characteristics) than in traditional work team environments. While we can confidently state that the relationship between team cohesion and team performance in non-ICE studies (e.g., firehouses, special operations teams) is positive and small to large, and similar to previous meta-analytic estimates (Beal et al., 2003), we cannot draw any conclusions about the direction and magnitude of the relationship between team cohesion and team performance in isolated and confined environments. Despite the limitations of such results, our findings highlight the importance of examining the effects of team cohesion on team performance in isolated and confined environments, and provide a cautionary note about generalizing findings from teams sometimes used as analogs that live and work together (non-ICE) to teams operating in isolated and confined environments. Similarly, limited information on other team factors (e.g., age homogeneity, education level homogeneity) and team performance inhibited us from estimating the true population validity of specific relationships in isolated and confined environments. Bringing further clarity to team cohesion for LDSEM, our figures that benchmarked team cohesion over time revealed that teams in shorter-duration missions spent more time with each other (an operationalization of team cohesion) than longer-duration teams. These results suggest limited usefulness of shorter-duration studies in understanding team cohesion for LDSEM.

As part of our quantitative review of team dynamics in LDSEM-analog environments, we also explored our benchmarking data set for trends in team dynamics over time (i.e., team efficiency, team conflict, team communication, team mood). Beginning with team efficiency, crews must coordinate and complete mission tasks in an efficient manner in order to achieve mission success (Salas et al., 2015a). Based on the available data, team efficiency in LDSEM-analog settings was relatively consistent across time; it was atypical for team efficiency to decrease over time. In uncommon situations in which team efficiency decreased during missions (see Vinokhodova et al., 2001; Eskov, 2011), researchers implicate ineffective role structure and conflict as possible triggers of the performance decrements (Sandal, 2001, 2004; Vinokhodova et al., 2001), suggesting that such factors are key threats to team efficiency. Further, the primary focus of team performance in LDSEM-analog environments has been efficiency. LDSEM will likely have team performance demands beyond team efficiency. For example, the team may need to be creative in order to use scare resources effectively, which suggests an expanded view of team performance in analog research is needed.

In contrast to team efficiency, intrateam conflict data greatly varied over time in LDSEM-analog settings, such that data do not show a consistent trend across teams. However, all teams reported at least one conflict within the team or with mission control by 40% of the mission completion or 90 days. Given that all teams engage in at least some conflict in extended mission, and will likely have to resolve these conflict incident rather autonomously, it is important to better understand conflict and effective conflict management strategies in LDSEM-analog settings.

With regard to team communication in LDSEM-analog settings, communication between crews and mission control is thought to provide valuable information about the psychological health of the crew and the interpersonal climate within the crew. It is interesting to note that one of the crews that demonstrated decreased efficiency over time (i.e., HUBES crew) also had shorter audio communication with mission control over time. Moreover, commanders' written communication with mission control across several missions were in line with the psychological closing phenomenon in that the length of commanders' reports to mission control decreased over time (Gushin et al., 1997, 2012). Analysis of communication is likely to provide a fruitful means for understanding team dynamics.

As for team mood—operationalized as total mood disturbance or positively affectivity—there was inconsistent support for the third quarter phenomenon (Steel, 2001; Dion, 2004; Kanas, 2004; Wang et al., 2014); however, three of seven LDSEM-analog teams reported an increase in negative affect in the third quarter of their missions. The two teams in particular that were studied for an extended period (i.e., greater than a year) both reported an increase in total mood disturbance approximately 1 year into the mission. These findings are important to note in light of the fact that team mood plays an instrumental role in team dynamics (e.g., Kahn and Leon, 2000; Steel, 2001). They suggests that it is prudent to better understand the effects of extended isolation on team mood for LDSEM.

### Limitations

The results described should be considered in light of the limitations of this research. In our attempt to quantitatively summarize team dynamics in LDSEM-analog environments, we were limited by the empirical research available within the extant literature (e.g., small sample size, correlational). The validity coefficients from the LDSEM-analog studies used in our analyses are based on small sample sizes. When weighted average analyses are based on smaller sample sizes, there is more uncertainty regarding how well an observed effect in a given sample reflects the true population validity. To help address this issue, based on the available data, we calculated improved estimates of the true population team predictor and team criterion relationships in an LDSEM-analog environment by inversely weighting the variances of the validity coefficients from the LDSEM-analog studies and the meta-analytic estimates of the same team predictor-criterion relationships from the extant literature. Additionally, we calculated the average inaccuracy of the estimates to generate 95% credible intervals regarding the uncertainty of the estimates. This approach afforded us the precision associated with meta-analytic estimates while accounting for the localness associated with a specific effect size from an LDSEM-analog environment.

Moreover, the studies included in our quantitative review were almost exclusively descriptive or correlational in design (see the work by Emurian and colleagues for a notable exception). With this is mind, we cannot make causal statements about the relationships examined in our review, nor can we disentangle the effects of one team predictor from another. Consequently, we encourage researchers to employ experimental and quasi-experimental designs to identify key threats to team dynamics and performance in LDSEM-analog settings. We acknowledge the limitations of this data (e.g., small sample size, correlational). Importantly, however, this is the data that we currently have for understanding team dynamics in LDSEM-analog environments.

### Future Directions for Research

Despite the limitations of this study, our findings provide insight into several potentially fruitful areas for research in regards to content, and research approaches related to extreme teams. In general, it seems that research should be prioritized when the nature of the relationship would be most likely to change as a function of the LDSEM context. One area in need of research is team affect. While most of the team mood data presented in this article were generated from aggregated individual-level data (for a notable exception see Šolcová et al., 2013), applying a team-level perspective and conducting investigations on team affect and team affect management could provide a more in-depth understanding of the role of affect in crew performance and crew member well-being. For example, team affect tends to become more homogenous through mechanisms such as emotional contagion (Totterdell et al., 1998), which could be magnified by specific characteristics of the LDSEM context (e.g., isolation and confinement). Also, crew composition factors (e.g., national diversity) could influence the emergence of team affect, norms for affective suppression or sharing, and the effectiveness of affect management approaches. Considering the unique features of ICE, further exploration of team affect, emotions, emotion regulation, and affect management in ICE across diverse crews and over time is warranted. Further, given that spikes in total mood disturbance were observed at the 1 year mark for studies in which teams both teams were in extended isolation, it is prudent to better understand the effects of extended isolation for LDSEM.

A second area in need of research is conflict management. LDSEMs provide a unique context in which conflict will need to be managed. Given the significant communication delays with those on Earth as teams travel into deep space, the teams will likely need to effectively manage conflict with at least some degree of autonomy. Our data suggest that at some point conflict is likely to occur between the crew, or between the crew and mission control. Indeed, LDSEMs are likely to be a situation where the crew will face competing or inconsistent priorities. For example, if more than one mission control is utilized for a particular mission, competing information may be given in regards to priorities (e.g., perform a function that requires the whole crew; require an individual adheres to a particular exercise schedule), which could create ambiguity in how crewmembers should allocate their time and resources. Crewmembers are likely to be diverse in a number of ways (e.g., professional, national background) which could also lead to misunderstandings or competing priorities (e.g., maintenance of the space vehicle, complete the science experiment) and potentially cause intra-team conflict (Bell et al., 2015a). The extent that crews effectively manage conflict will be of great importance given the expected durations of the space missions, the inability for crewmembers to leave, and the limited and delayed communication with mission control possibly compounding issues between the team and mission control. A better understanding of conflict and the conflict management cycle as teams live and work together in extended isolation and confinement is prudent.

In addition to their effects on team performance, conflict management and affect are important areas for future research because they will likely play a critical role in a team's resilience. While researchers are working diligently to mitigate all potential threats to team effectiveness, LDSEM crews will inevitably face challenges. A key aspect of correctly composing, training, and providing countermeasure support to crews will include consideration of the crew's resilience, defined as the capability to withstand and recover from stressors, pressure, or challenges (Alliger et al., 2015). Crewmembers' challenges may range from subtle changes that result in a less than ideal team state (e.g., the general decline in positive mood) to events that are more acute in nature (e.g., dispute related to the involvement of MC in conflict management). Regardless of the specific challenge, team resilience will likely be critical to the success of crews on LDSEMs. Future research should examine the effects of specific manipulations of stressors on crew resilience as well as the effects of subtle changes that occur during a team's life cycle on crew resilience.

We believe the decline in team efficiency during the HUBES simulation and the dip in team efficiency for one of the teams during the SFINCSS simulation provide interesting directions for future research in LDSEM-analog settings. Several researchers (e.g., Sandal, 2001; Vinokhodova et al., 2001) suggested that the decline might have been due to intra-team conflict and instability in or a lack of established leadership structure. Given the autonomy of the crew at long-distances from Earth, and the likelihood that crews will include individuals from both high and low power distance countries, a better understanding of the conditions needed for teams to establish a workable leadership structure, and the process for ensuring crews high in gender and cultural diversity can effectively resolve status conflict is necessary (Bendersky and Hays, 2012).

Finally, a number of methodological recommendations can be made for future research. First, sample sizes in high fidelity environments to LDSEM, particularly ICE, are likely to be small. Where possible, data should be collected in such a way that they can be aggregated and compared across multiple studies. Ideally, enough data should be collected to generate an effect size. The normality of the data could be reported (or even better, the raw data) to allow future summaries to ensure the data are being appropriately modeled. When the sample size is too small to allow an effect size to be generated, data on key team constructs (e.g., team efficiency, communication, mood, and cohesion) should be collected with a common set of measures. Analog research on mood has consistently relied on the PANAS and POMS which made comparisons across studies possible. Researchers at the Russian Academy of Science's Institute of Biomedical Problems and some individual researchers (e.g., Leon) have consistently collected data using the same measures, which allowed us to report many of the figures in this article. In addition, NASA's Human Research Program is adopting a standardized set of measures to be collected across NASA analogs that includes measures such as team conflict, team cohesion, and team mood as well as other constructs. For key constructs (e.g., conflict, mood, cohesion), it is essential that analog research use the same measures so that the data better lend itself to the eventual culmination of studies.

Second, continued research is needed on small sample sizes. As an example, some meta-analytic approaches (e.g., Bayesian, Fisherian) calculate sample variance as 1/*n* and others as 1/(*n*-3) (Brannick, 2001), and the Schmidt and Hunter (2015) method uses *n*-1 in the denominator of their random effects meta-analysis of correlations. As Brannick (2001) states, “if the sample is so small that the choice of *n* or *n*-3 is critical, then the researcher has a more serious issue to confront, namely, how to collect more data” (p. 469). Unfortunately for analog researchers, more data is not likely to be a feasible option for many studies. While differences in how sampling variance is calculated and the ability to calculate sampling variance at small sample sizes may generally be less of an issue in traditional meta-analyses, it is an important issue for the eventual culmination of team LDSEM-analog research. Future research may wish to explore the accuracy of the different meta-analytic approaches for use with extremely small sample sizes (e.g., correlations based on 3 to 7 teams) through simulations as well as develop alternative versions of quantitative aggregation for small sample sizes. Continued advances in analytics that can best represent small sample size data is likely to be important for space research as well as extreme teams in general (Bell et al., 2018).

## Conclusions

Future space exploration teams will be required to work effectively under complex and dangerous conditions to successfully accomplish their missions. With an understanding of team dynamics in LDSEM-analog environments, we can minimize potential threats to mission success while optimizing team performance. While an extensive research base exists that examines teams in LDSEM-analog environments, small sample sizes make traditional forms of meta-analysis inappropriate. Importantly, however, this is the data that we have for understanding team dynamics for future LDSEMs. Given this, we used a weighted averages approach to generate minimum variance estimates of team predictor and outcome relationships, and generated descriptive figures depicting team dynamics over time. Our systematic review of quantitative research on teams in LDSEM-analog settings summarizes what we know about team dynamics for future LDSEM, and provides guidance for future research.

## Author Contributions

STB developed the analytic approach, designed the search process and coding forms, conducted data analyses, and wrote aspects of the manuscript. SGB and TM conducted extensive literature searches, coded articles included in the review, generated tables and figures, and wrote aspects of the manuscript.

### Conflict of Interest Statement

The authors declare that the research was conducted in the absence of any commercial or financial relationships that could be construed as a potential conflict of interest.
