# Supplementary material for: What We Know About Team Dynamics for Long-Distance Space Missions: A Systematic Review of Analog Research
Source: Front Psychol. 2019 May 15;10:811. doi: 10.3389/fpsyg.2019.00811 (PMC6530432; doi:10.3389/fpsyg.2019.00811)
Supplement: Supplementary file 1 [file Table_1.DOCX]

Table 1.

*Fidelity of Data Sets to LDSEM*

| Data Set | *k* | Study Setting | Publication Year | Mean Crew Size | Summary Demographics | Familiarity | Dangerous Environment | Mission Length (Days) |
| --- | --- | --- | --- | --- | --- | --- | --- | --- |
| Effect Size | 11 | Natural analogs (4); military/firehouse (6); lab studies (1) | 50% published pre-1985 | Range: 4-18; 1 team of 40 | Mean age: late 20s; mostly men; similar professional background | NR | Approx. 50% operated in dangerous environments | Range: 10-730 |
|  |  |  |  |  |  |  |  |  |
| Benchmark | 61 | Space simulations (17); natural analogs (21); lab studies (5); ISS missions (2) | 70% published post-2000 | Range: 2-28; 75% had 6 members or fewer | Age range: 27-36; approx. 75% of teams were all or mostly men; approx. 50% reported some professional diversity; approx. 40% reported diversity on national background | 45% of articles reported some familiarity among members | Approx. 30% operated in dangerous environments | Range: 2-520 |
| *Note:* NR = Data not reported. | | | | | |  |  |  |
