# Supplementary material for: What We Know About Team Dynamics for Long-Distance Space Missions: A Systematic Review of Analog Research
Source: Front Psychol. 2019 May 15;10:811. doi: 10.3389/fpsyg.2019.00811 (PMC6530432; doi:10.3389/fpsyg.2019.00811)
Supplement: Supplementary file 3 [file Table_3.docx]

Table 3.

*Analog Data Sources and Meta-analytic Effects Contributing to the Weighted Averages*

Note. Hmgnty = Homogeneity. Trnsfrm Ldrshp = Transformational Leadership. *n* = sample size of the analog study; *r* or rank = effect size taken from the analog study, or a linear composite or relevant effect sizes from the study; ρ_local_= *r* from the analog study corrected for predictor and criterion unreliability; ρ_prior_ = meta-analytic mean corrected correlation (estimate chosen was corrected for predictor and criterion unreliability);
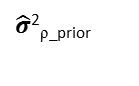
= the estimate from the meta-analysis of the true population variability in effect sizes; *k* = the number of primary studies included in the meta-analysis; *n_meta_* = average sample size of a primary study included in the meta-analysis.
